# Grapevine Shoot Extract Rich in *Trans*-Resveratrol and *Trans*-ε-Viniferin: Evaluation of Their Potential Use for Cardiac Health

**DOI:** 10.3390/foods12234351

**Published:** 2023-12-02

**Authors:** María del Mar Contreras, Anouar Feriani, Irene Gómez-Cruz, Najla Hfaiedh, Abdel Halim Harrath, Inmaculada Romero, Eulogio Castro, Nizar Tlili

**Affiliations:** 1 Department of Chemical, Environmental and Materials Engineering, Campus Las Lagunillas, Universidad de Jaén, 23071 Jaén, Spain; igcruz@ujaen.es (I.G.-C.); iromero@ujaen.es (I.R.); ecastro@ujaen.es (E.C.); 2 Centre for Advanced Studies in Earth Sciences, Energy and Environment (CEACTEMA), Campus Las Lagunillas, Universidad de Jaén, 23071 Jaén, Spain; 3 Laboratory of Biotechnology and Biomonitoring of the Environment and Oasis Ecosystems, Faculty of Sciences of Gafsa, University of Gafsa, Gafsa 2112, Tunisia; ferianianwer@yahoo.fr (A.F.); najhfaiedh@yahoo.fr (N.H.); 4 Department of Zoology, College of Science, King Saud University, Riyadh 11451, Saudi Arabia; hharrath@ksu.edu.sa; 5 Institut Supérieur des Sciences et Technologies de l’Environnement, Université de Carthage, Hammam Chat 2050, Tunis 1073, Tunisia; nizar.tlili@fst.rnu.tn

**Keywords:** cardiac health, grapevine, shoots, stilbenoids, *trans*-resveratrol, valorization

## Abstract

A grapevine shoot extract (GSE) was obtained using ultrasound-assisted extraction and characterized. The main phenolic constituents were identified as stilbenoids. Among them, *trans*-resveratrol and *trans*-ε-viniferin stood out. The GSE was administered to an isoproterenol-induced myocardial injury animal model. The extract alleviated the associated symptoms of the administration of the drug, i.e., the plasma lipid profile was improved, while the disturbed plasma ion concentration, the cardiac dysfunction markers, the DNA laddering, and the necrosis of myocardial tissue were diminished. This effect could be related to the anti-oxidative potential of GSE associated with its antioxidant properties, the increased levels of endogenous antioxidants (glutathione and enzymatic antioxidants), and the diminished lipid peroxidative markers in the heart. The results also revealed angiotensin-converting enzyme (ACE)-inhibitory activity, which indicated the potential of GSE to deal with cardiovascular disease events. This work suggests that not only *trans*-resveratrol has a protective role in heart function but also GSE containing this biomolecule and derivatives. Therefore, GSE has the potential to be utilized in the creation of innovative functional ingredients.

## 1. Introduction

In 2022, the world vineyard surface was about 7.3 million hectares, a value that seems to have remained stable since 2017. It was dedicated to vines for wine, juice, table grapes, and dried grapes [1]. For vineyard management, pruning is aimed to ensure optimal grape production and quality [2]. In this practice, grapevine shoots are generated as waste, whose current management (habitual burning in the field) generates environmental impact [3]. By incorporating grapevine shoots into a circular bioeconomy framework, vineyards and related industries can contribute to resource efficiency, waste reduction, and sustainable practices that benefit both the environment and the economy.

A potential means of valorization could be the extraction of bioactive compounds, including stilbenoids, which are health-promoting compounds. They can find food applications (e.g., as preservatives and functional ingredients), medical applications, and uses as vine biostimulants [4]. Among the stilbenoids, resveratrol stands out, but it presents a high variation in this type of biomass; in some samples, this compound was not detected [5], while in others its concentration can reach about 7 g per kg of biomass [6]. Some factors affecting this variation are the genotype (or the cultivar type), climate, agronomic practices, and stress-related factors [6]. Particularly, grapevine shoots of traditional and non-traditional cultivars have been evaluated, for example, from Spain [5,7], Portugal [8], Italy [9], and France [10]. Other cultivars, like the Spanish white grape cultivar ‘Charelo’ (or Xarel·lo), have been poorly studied.

The extraction conditions can also affect the phenolic profile and yield [3,8] and hence they should be jointly evaluated with other factors such as the cultivar. For this purpose, several works described the application of green technologies to provide efficient extraction, e.g., high-voltage electrical discharge [11], microwave [5,12], subcritical water [12], and ultrasound [5]. The latter technology has shown good extraction performance both in the laboratory and at the pilot scale, highlighting its suitability for industrial application [8].

In this context, the main aim of the work was to characterize and evaluate the bioactive potential of a grapevine shoot extract (GSE) obtained from the cultivar ‘Charelo’ using ultrasound-assisted extraction (UAE). First, the phenolic composition was further characterized. Other compounds (sugars, alditols, and organic acids) were also quantified to provide more information about the overall composition of the extract. Second, the efficacy of the GSE on an isoproterenol (ISP)-induced heart injury model was studied. Although there is evidence of the protective role of *trans*-resveratrol on cardiovascular and heart health [13], this work brings new insights into the potential use of natural extracts containing *trans*-resveratrol and *trans*-ε-viniferin to provide functional ingredients for the food, pharma, and nutraceutical sectors.

## 2. Materials and Methods

### 2.1. Raw Biomass and Extraction

Grapevine shoots were obtained from a local company in the Penedés region (northeast of Spain) and air-dried to about 8% humidity. The sample was milled to ~1 mm with an ultracentrifugal ZM 200 mill (Retsch GmbH, Haan, Germany). Then, the sample was extracted under previously optimized conditions using 10% solids (*w*/*v*) suspended in 80% ethanol aqueous solution and ultrasonicated for five min at 80% amplitude with a Sonifier SF550 (Branson, L’Hospitalet de Llobregat, Spain). The extraction was initiated at room temperature (~20 °C) and monitored at the end of the experiments (57 °C). The extract was finally freeze-dried (Noxair freeze-drier, Barcelona, Spain). This extract (GSE) was further characterized and administered to the animal model.

Moreover, maceration for 24 h was applied as control using the same solid-to-liquid ratio (10%, *w*/*v*) and solvent (80% ethanol) at room temperature and aided by agitation at 150 rpm in an incubation shaker (InforsHT Ecotron, Surrey, UK).

### 2.2. Characterization of the Extract

#### 2.2.1. Phenolic Content, Composition, and Antioxidant Assays

GSE was redissolved in 80% (*v*/*v*) ethanol for analysis. The total phenolic content (TPC) was measured by a colorimetry test using Folin and Ciocalteu’s phenol reagent in microplates from Sigma-Aldrich (St. Louis, MO, USA) in a Bio-RadiMark™ device (Hercules, CA, USA), according to a previous study [14]. The antioxidant activity was determined using the ferric reducing power (FRAP) at 734 nm in the aforementioned device [14]. Commercial gallic acid (TPC) and Trolox (FRAP), both from Sigma-Aldrich, were used for comparison [14].

The phenolic profiles were acquired using two methodologies, one using high-performance liquid chromatography (HPLC) and UV detection and the other based on HPLC–quadrupole time-of-flight (QTOF)–mass spectrometry (MS), according to a previous study [15]. The former analysis was performed in a Prominence UFLC chromatograph (Shimadzu Corporation, Kyoto, Japan) using a BDS HYPERSIL column (C18 with 5 μm particle size; dimensions, 4.6 mm × 250 mm) (Thermo Fisher Scientific Inc., Waltham, MA, USA). The gradient conditions and mobile phases were in accordance with previous studies [14,15]. The commercial standards of *trans*-resveratrol and *trans*-ε-viniferin were obtained from Extrasynthese (Genay, France) and used for quantification in the aforementioned chromatographic conditions at 280 nm. The curves were *y* = 55,984*x* + 347,526 (R^2^ = 0.992) for *trans*-resveratrol and *y* = 28,962*x* + 315,646 (R² = 0.998) for *trans*-ε-viniferin.

The RP-HPLC-QTOF-MS analysis was conducted in an Agilent 1200 HPLC connected online to an Agilent 6530B QTOF mass analyzer through an electrospray interface using the negative ionization mode (Agilent Technologies, Santa Clara, CA, USA). A C18 column (a core-shell Kinetex with 2.7 µm particle size; dimensions, 2.1 mm × 50 mm) (Phenomenex, Barcelona, Spain) was used by applying a gradient with 0.1% *v*/*v* formic acid and acetonitrile [15]. The mass formula was generated with a mass error lower than 5 ppm for characterization purposes using an Agilent MassHunter Qualitative Analysis B.06.00 (Agilent Technologies). The peak area of the ions was obtained to estimate the relative abundance.

#### 2.2.2. Other Components

The carbon, hydrogen, nitrogen, and sulfur contents of the GSE were obtained via combustion of about 2 mg of sample in a TruSpec Micro device (LECO corporation; St. Joseph, MI, USA). The protein content was estimated as the nitrogen content × 6.25.

The lyophilized GSE was dissolved in ultrapure water for the subsequent analyses and analyzed following a previous study [14]. The sugar content was quantified using a Waters Prostar HPLC device with refractive index detection (RID) (Milford, MA, USA) [14]. Moreover, the content of organic acids was quantified with HPLC–RID in an Agilent HPLC 1260 series device (Agilent Technologies). The columns were CARBOSep CHO-782 Pb and ICSep ICE-COREGEL 87 H3 from Transgenomic, Inc. (Omaha, NE, USA) heated at 70 °C and 65 °C, respectively. The mobile phases were ultrapure water and ultrapure water with sulfuric acid (5 mM) flowing at 0.6 mL/min, respectively [14].

### 2.3. In Vivo Evaluation of the Cardiac Protective Activity

#### 2.3.1. Animals

The current investigation was granted approval by the Ethical Committee for the Care and Use of Laboratory Animals (reference no. FSG-04-23; University of Gafsa, Tunisia). Forty-eight Wistar male rats, with a body weight (bw) of about 230–250 g, were purchased from SIPHAT (Ben Arous, Tunisia). They were kept housed with unrestricted access to water and food, and the light (12 h), temperature (22–24 °C), and humidity (50%) were controlled.

#### 2.3.2. Acute Preliminary Toxicity Effect

GSE in saline was administered orally to the rats at 5, 10, 20, 40, and 60 mg GSE/kg bw doses, with six animals in each group. For comparison, the treated animals and untreated rats (*n =* 6) were carefully observed for any toxicological symptoms within 24 h and during a subsequent 21-day observation period.

#### 2.3.3. Experimental In Vivo Assay

The rats were adapted for 15 days to the aforementioned conditions and then split into six groups (*n =* 6, per group). The duration of the experiment and treatments was 30 days as follows:

Groups I (C): Control rats that were not subjected to any treatment, received daily doses of saline solution (1 mL each dose).

Groups II (GSE1): rats were treated with 1 mL of saline solution containing GSE (20 mg GSE/kg bw) for 28 days. The administration was carried out by gavage.

Groups III (GSE2): rats were treated with 1 mL of saline solution containing GSE (40 mg GSE /kg bw) for 28 days. The administration was carried out by gavage.

Groups IV (ISP): ISP in saline was injected (85 mg/kg bw) for two successive days (on the 29th and 30th days). Saline solution was administered daily for 28 days before injection of ISP.

Groups V (GSE1 + ISP): 1 mL of saline solution containing GSE (20 mg GSE/kg bw) was administered daily by gavage for 28 days, and ISP in saline was then injected on the 29th and 30th days.

Groups VI (GSE2 + ISP): 1 mL of saline solution containing GSE (40 mg GSE/kg bw) was administered daily by gavage for 28 days, and ISP in saline was then injected on the 29th and 30th days.

#### 2.3.4. Electrocardiogram

The electrocardiographic test was realized with an ECG VET 110 electrocardiograph (Biocare, Shenzhen, China) after isoproterenol was injected (30th day) and before the sacrifice. Rats received anesthesia using ketamine hydrochloride (100 mg/kg bw) intraperitoneally and needle electrodes were non-invasively inserted in the lead II position after 15 min.

#### 2.3.5. Biological Sample Collection

Blood from rats was collected in ethylenediaminetetraacetic acid (EDTA) just after being sacrificed by cervical decapitation. The blood was subjected to 10 min centrifugation at 3500 rpm at 4 °C to recover the plasma. The hearts were collected, weighed, and washed. To determine the myocardial infarction (MI) area, some fragments of the hearts were fixed using 1% 2,3,5-triphenyltetrazolium chloride (TTC) in phosphate-buffered saline for 15 min. For histopathological analysis, other fragments of the hearts were fixed using 10% buffered formaldehyde. The remaining fragments were maintained at −70 °C for further studies.

#### 2.3.6. Analysis of Cardiac Parameters in Plasma

Various commercial kits from Zellbio (Lonsee, Germany) were applied to determine the concentration of aspartate aminotransferase (AST), lactate dehydrogenase (LDH), fibrinogen, and creatine kinase-MB (CK-MB). Moreover, the cardiac troponin (cTn-I) amounts were estimated using a Roche Diagnostics immunoassay based on electrochemiluminescence (Basel, Switzerland). The activity of angiotensin-converting enzyme (ACE) was calculated using standard kits (Trinity Biotech’s, Bray, Ireland).

LDL-cholesterol (LDL-C), HDL-cholesterol (HDL-C), triglycerides (TG), and total cholesterol (TC) amounts were calculated according to the Biomaghreb kit’s manufacturer (Tunisia).

The concentration of electrolytes (Na^+^ and Ca^2+^) was determined using an EasyLyte Plus ionogramme analyzer (Medica; Bedford, MA, USA).

#### 2.3.7. Determination of Cardiac Pro-Antioxidant and Antioxidants Levels

Oxidative stress was estimated in the heart tissue homogenate through the measurement of thiobarbituric acid reactive substances (TBARS) to express the nmol malonaldehyde (MDA)/g tissue following the reported methodology [16].

The endogenous antioxidant potential was estimated by measuring the level of activity of superoxide dismutase (SOD) and catalase (CAT) according to published protocols [17,18], respectively, and cellular glutathione (GSH) determination was performed according to a previous method [19], and according to Chtourou et al. [20]. The protein content was quantified following Bradford’s study [21].

#### 2.3.8. DNA Fragmentation Analysis

DNA from the hearts of animals was extracted using the methodology previously reported [20]. The obtained DNA was stained using ethidium bromide and placed on agarose gel for electrophoresis (0.8%), which was observed and UV photographed for interpretation.

#### 2.3.9. Determination of Heart Pro-Apoptotic Genes

The apoptosis event was inspected in the myocardial tissue using real-time (RT)-PCR assays of three genes (B-cell lymphoma-2 associated-x or Bax, B-cell lymphoma-2 or Bcl2, and caspase-3) following Chtourou and colleagues’ study [20]. Briefly, 2 µg of isolated mRNA was reversely transcribed using the Invitrogen superscript reverse transcriptase. The amplification was performed by applying previously described RT cycler conditions [22] and the primer sequences described in Table S1 for the RT-PCR. Ethidium bromide was used for staining the PCR products after electrophoresis on 1.8% agarose gel. Finally, the software ImageJ 1.54f (NIH, MD, USA) was used to determine the mRNA expressions of the genes.

#### 2.3.10. Infarct Size Determination

The hearts were fixed in 1% TTC according to a previously reported method [23] (Section 2.3.5.). The color red indicated normal myocardial tissue, while a pale orange suggested damaged areas.

#### 2.3.11. Histopathological Analysis

Hematoxylin-eosin (H&E) staining was used on 5 μm cut sections from paraffin blocks [23], which were then analyzed under light microscopy and photographed.

### 2.4. Statistical Analysis

Data from the characterization represent the mean or the mean ± standard deviation (SD) of three analyses. Data from in vivo experiments represent the mean ± SD (six rats/group) and they were analyzed via a one-way analysis of variance and Tukey’s test (*p* < 0.05 for statistical significance) with GraphPad Prism 4.02 (Boston, MA, USA).

## 3. Results

### 3.1. Phenolic Composition and Antioxidant Potency

**Figure 1:**
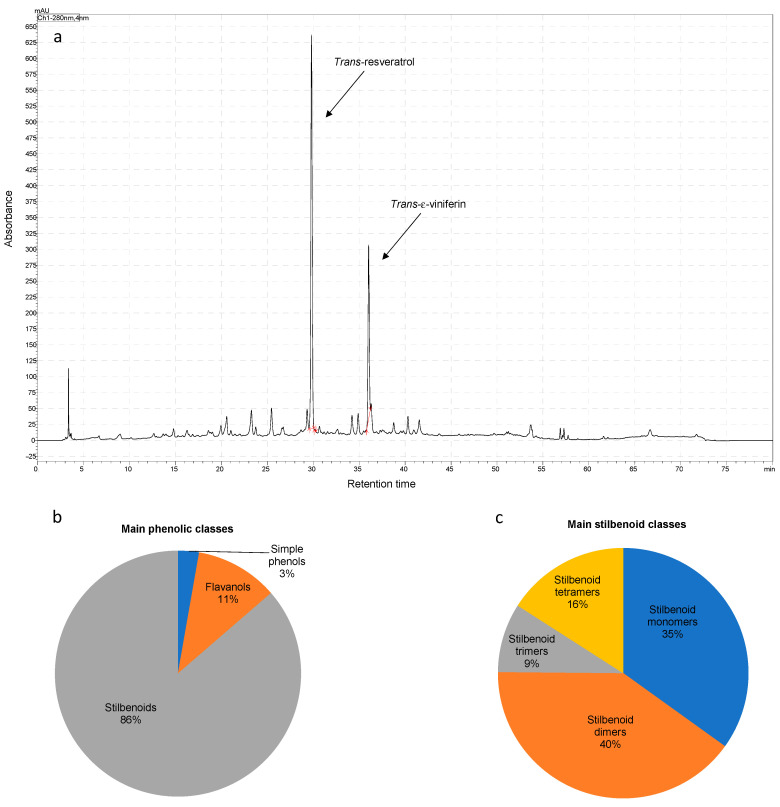
(**a**) UV chromatogram of the vine shoot extract at 280 nm. (**b**) Distribution of phenolic classes and (**c**) stilbenoids using the relative area obtained by RP-QTOF-MS. Simple phenols refer to gallic acid and phenol aldehydes.

The TPC and the antioxidant potential of the GSE, which was obtained by UAE, are shown in Table 1. The TPC was 207.3 g/g GSE, which is about 8 mg/g grapevine shoots (on a dry basis). The main phenolic compounds obtained by UAE from the grapevine shoots were *trans*-resveratrol and *trans*-ε-viniferin, as suggested by the HPLC-UV analysis at 280 nm (Figure 1). These compounds were quantified for the standardization of the GSE, highlighting that they contained about 27.4 ± 0.3 mg/g GSE and 29.2 ± 0.2 mg/g GSE, respectively.

The extract was also further characterized using QTOF-MS-based analysis. The compounds were tentatively characterized based on the retention time, generated molecular formula, and literature on phenolic compounds found in grapevine plant parts and wine-associated waste (particularly, [6]). The results are shown in Table 2, which depicts that 27 phenolic compounds were found globally. They were classified as stilbenoids, which were distributed as four monomers, nine dimers, two trimers, and five tetramers, simple phenolic compounds (a hydroxybenzoic acid, gallic acid, and two phenol aldehydes), three monomeric flavanols, and a B-type dimeric flavanol. Figure 1b illustrates the overall distribution of phenolic compounds based on the relative area measured analysis by RP-HPLC-QTOF-MS. This semi-quantitative analysis suggested that GSE was rich in stilbenoids in monomeric and dimeric forms (Figure 1c), with *trans*-resveratrol and its dimer *trans*-ε-viniferin prominent among them, as detected by UV.

### 3.2. Other Components of GSE

The elemental analysis, including the carbon, hydrogen, nitrogen, and sulfur contents of the GSE, is shown in Table 3, along with other components that could be characterized in the extract, i.e., carbohydrates in monomeric and oligomeric forms, alditols (mannitol), and organic acids (acetic and formic acids). These results suggest that overall, the extract contained about 9% moisture and consisted of about 20% TPC, 11% glucose carbohydrates, 0.3% galactose carbohydrates, 3% mannitol, 4% organic acids, 7% crude protein, and 6% inorganic components, determined as ash.

### 3.3. Preliminary Acute Toxicity Evaluation

Using the tested doses of GSE, no alterations in the behavior were noted, and the mortality of the animals remained unchanged up to 60 mg GSE/kg bw.

### 3.4. Effect of GSE on Rats’ Weight

The results relative to rats’ body and heart weight and CWI are presented in Table 4. The studied groups did not exhibit any substantial changes in body weight. Nevertheless, the highest CWI (*p* < 0.05) was observed in the ISP group. It was also remarkable that GSE1 or GSE2 pre-co-treatment induced a significant decrease in relative heart weight in comparison with the ISP group.

### 3.5. Effect of GSE on ST-Segment

In comparison with the normal group C, the results showed that the ISP group presented an elevated ST-segment (Pardee wave) which is related to MI (Figure S1). The pre-co-treatments with GSE1 or GSE2 reduced the ECG perturbation by the decrease in the ST-segment by comparison with the animals that solely received ISP. It was also remarkable that GSE2 was more effective than GSE1 against ISP administration.

### 3.6. Effect of GSE on the Cardiac Indicators of Injury

**Figure 2:**
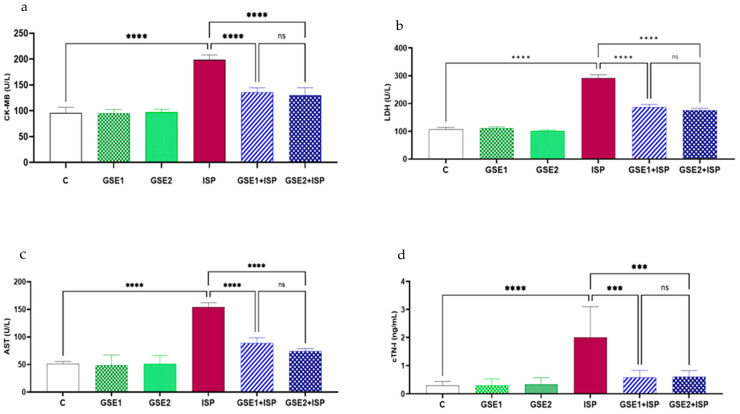
Plasma activities of (**a**) creatine kinase-MB (CK-MB), (**b**) lactate dehydrogenase (LDH), (**c**) aspartate aminotransferase (AST), and (**d**) cTn-I levels in normal and treated rats (mean ± SD; *n =* 6 rats per group). C, control; GSE, grapevine shoot extract; ISP, isoproterenol. **** *p* < 0.0001: ISP vs. C; *** *p* < 0.001 or **** *p* < 0.0001: GSE1 + ISP/GSE2 + ISP vs. ISP; ns (not significant): GSE1 + ISP vs. GSE2 + ISP.

The activities of AST, CK-MB, LDH, and cTn-I associated with the cardiac injury of all groups are shown in Figure 2. The results showed that the activities of the targeted markers were higher in the ISP group than in the control rats. GSE1 or GSE2 pre-co-treatment remarkably attenuated the effect of ISP on the studied enzymes and decreased their activities, when compared to ISP-treated animals. No substantial variations in these markers were noted among the GSE1-ISP and the GSE2-ISP rats.

### 3.7. Effect of GSE on Fibrinogen Concentration

**Figure 3:**
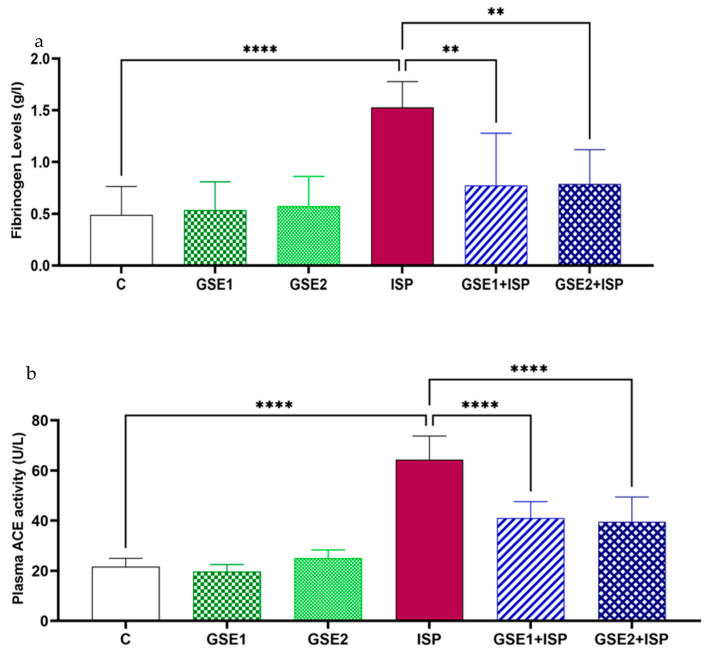
Changes in the (**a**) fibrinogen levels and (**b**) angiotensin-converting enzyme (ACE) activity in the plasma of different animal groups (mean ± SD; n= 6 rats per group). C, control; GSE, grapevine shoot extract; ISP, isoproterenol. **** *p* < 0.0001: ISP vs. C; ** *p* < 0.01 or **** *p* < 0.0001: GSE1 + ISP/GSE2 + ISP vs. ISP.

As Figure 3a shows, the concentration of plasmatic fibrinogen in the ISP-treated animals increased in comparison with the control group, at *p* < 0.0001. This figure also shows that the pre-co-administration of GSE diminished the fibrinogen concentration at *p* < 0.05 in contrast with the ISP group.

### 3.8. Effect of GSE on ACE

Figure 3b displays the activity of ACE in the studied animals. Rats treated with ISP presented a high activity of ACE in comparison with the control group (*p* < 0.0001). Alternatively, the association of ISP with GSE1 or GSE2 remarkably decreased (*p* < 0.05) the enzyme activity compared to the aforementioned group.

### 3.9. Effect of GSE on Plasmatic Lipids

The lipid profile was significantly disrupted by ISP as observed in the ISP group plasma (Table 5). It was clear that ISP increased the TC, TG, and LDL-C amounts, and decreased the HDL-C level at *p* < 0.05. The administration of ISP with GSE1 or GSE2 restored the concentration of lipid markers to normal levels (*p* < 0.05).

### 3.10. Effect of GSE on Major Plasma Electrolytes

Table 5 also depicts the impact of the studied interventions on the concentrations of electrolytes in plasma. In contrast to the control animals, the obtained data suggested that the administration of ISP notably enhanced (*p* < 0.05) the amounts of Ca^2+^ and diminished the concentration of Na^+^. However, rats subjected to the combined treatment with GSE1 or GSE2 showed levels of targeted electrolytes near normal values detected in control groups.

### 3.11. Effect of GSE on MDA, CAT, SOD, and GSH Levels

The levels of MDA together with the activity of SOD and CAT, as oxidative stress biomarkers in the cardiac tissue, are shown in Table 5. The results suggest that the administration of ISP elevated the amounts of MDA and jointly decreased the activities of the targeted enzymes, as well as the GSH level, compared to normal rats. The levels and the activities of the studied biomarkers were significantly restored in the animals pre-co-treated with GSE1 or GSE2 (*p* < 0.05).

### 3.12. Effect of GSE on the DNA from Cardiac Tissue

**Figure 4:**
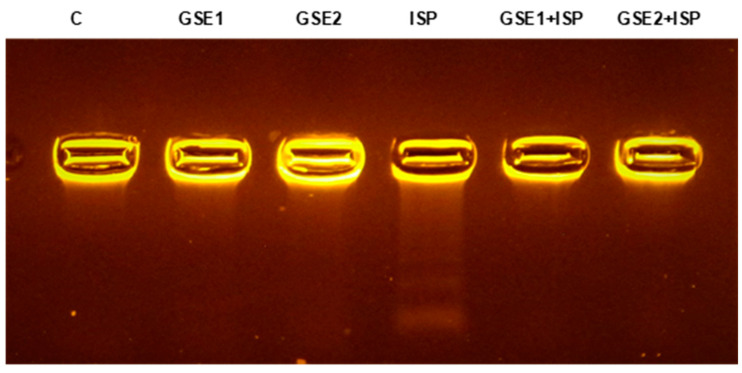
DNA electrophoresis on agarose gel after extraction from the heart tissue of the control group (C) (lane 1) and animals treated with grapevine shoot extract (GSE), GSE1 (lane 2) and GSE2 (lane 3), isoproterenol (ISP) (lane 4), and with GSE and ISP, GSE1 + ISP (lane 5), and GSE2 + ISP (lane 6).

The effect of the different treatments on the integrity of genomic DNA from cardiac tissue is illustrated in Figure 4. An intact band was observed in the DNA electrophoretic gel of control rats (lane 1). Significant DNA damage was induced by ISP treatment, resulting in DNA fragmentation showing a mixture of smearing and laddering (lane 4). On the other hand, the pre-co-administration with GSE1 or GSE2 (lanes 5 and 6, respectively) decreased the observed ISP-induced genotoxicity (Figure 4).

### 3.13. Effect of GSE on Pro-Apoptotic Genes

**Figure 5:**
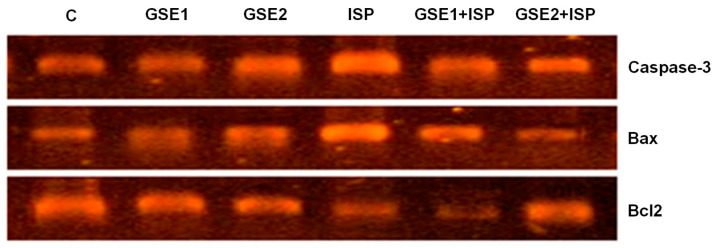
Agarose gel electrophoresis of caspase 3, Bax, and Bcl2 genes in the heart tissue of the control group (C) (lane 1) and animals treated with grapevine shoot extract (GSE): GSE1 (lane 2) and GSE2 (lane 3), isoproterenol (ISP) (lane 4), and with GSE and ISP, GSE1 + ISP (lane 5), and GSE2 + ISP (lane 6).

Figure 5 presents the cardiac mRNA expression of caspase-3, Bax, and Bcl2 in the studied animals. In the ISP group, the results showed a remarkable up-regulation of pro-apoptotic biomarkers caspase-3 and Bax compared to the untreated group. The pre-co-administration with GSE1 or GSE2 significantly reduced the observed overexpression associated with ISP. Alternatively, a down-regulation of the Bcl2 gene was observed for the ISP group, which was especially reverted in the GSE2-treated rats.

### 3.14. Infarct Size Staining

**Figure 6:**
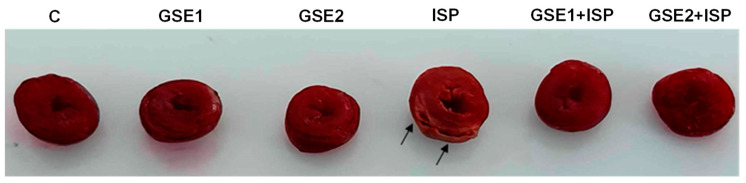
Myocardial tissue from the studied animal groups after TTC staining and photomicrography. C, control; GSE, grapevine shoot extract; ISP, isoproterenol. Arrow: infarcted areas.

The myocardial tissue images and infarct size are presented in Figure 6. The control group tissue displayed a normal structure, which was indicated by red color using TTC. Animals that received ISP displayed large necrotic and infarcted areas, identifiable by a pale orange color. It was remarkable that the pre-co-treatment using GSE decreased the infarct size in comparison to MI rats induced by ISP (Figure 6).

### 3.15. Effect of GSE on the Histology of the Heart Tissue

**Figure 7:**
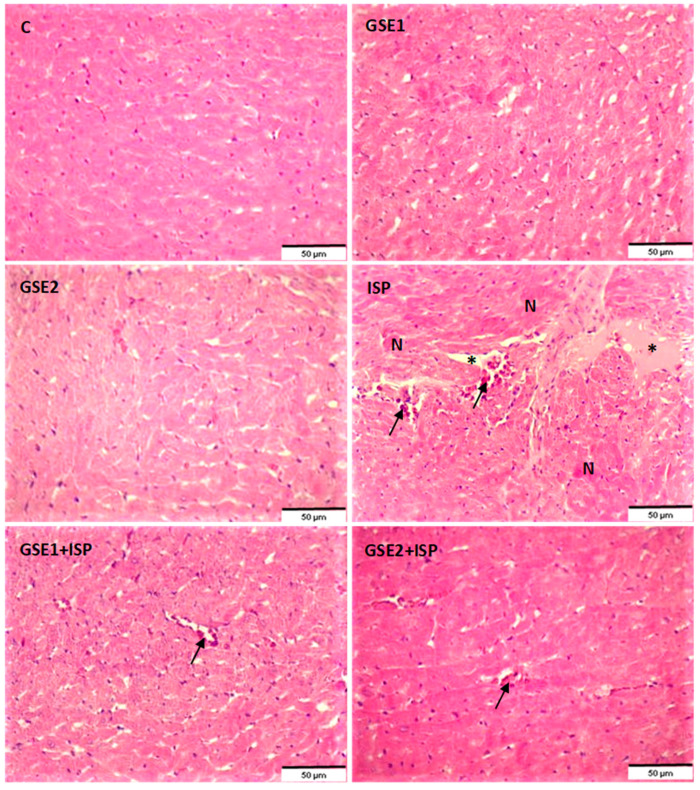
Histological images of heart tissue of the control and treated groups using hematoxylin and eosin staining at ×200 magnification. C, control; GSE, grapevine shoot extract; ISP, isoproterenol. Arrow: inflammatory cell infiltration; asterix: separation of cardiac myofibrillar; N: myocardial cell necrosis.

The histopathologic modification was studied using fixed cardiac slices and H&E staining in all studied groups (Figure 7). The H&E staining of cardiac tissues from the normal group (C) showed several multinucleated myofibers and myocyte necrosis was not observed. The heart tissue of ISP-treated rats showed histopathologic changes as manifested by excessive leukocyte infiltration, myocardial cell necrosis, and separation of cardiac myofibrillar. In contrast with this group, the pre-co-administration with GSE1 reduced the levels of inflammatory cells, and a moderate disturbance of myofibers and little muscle separation was observed (GSE1 + ISP). Moreover, lower myonecrosis and reduced infiltration of inflammatory cells were observed in the GSE2 + ISP group.

## 4. Discussion

The valorization of grapevine shoots is crucial to promote circularity in the grape and wine sectors. Previous studies have identified stilbenoids in grapevine plant parts of various grapevine cultivars and *Vitis* species [10,12,24], which are relevant biomolecules to produce bio-based products, including functional ingredients. However, their composition and content are highly variable depending on the cultivar, studied part, and extraction conditions. Therefore, in this work, grapevine shoots from the cultivar ‘Charelo’, which have been poorly studied, were extracted using maceration aided by agitation and ultrasound using the same solvent (80% *w*/*v* ethanol) and solid loading. The liquid extracts were analyzed. Preliminarily, it was evidenced that the application of ultrasound required lower energy consumption compared to maceration (0.009 kW h vs. 0.490 kW h, respectively) while increasing the solubilization of the total phenolic content (7.8 mg/g vs 6.5 mg/g of grapevine shoot, respectively). Moreover, ethanol was selected as the extraction agent since it is safer than other organic solvents from the health and environmental points of view to promote sustainable processes [25]. Therefore, the use of UAE with 80% ethanol seems promising for recovering phenolic compounds from grapevine shoots.

The extract obtained by UAE was lyophilized (GSE) and further characterized. The GSE mainly contained stilbenoids, highlighting the presence of *trans*-resveratrol and *trans*-ε-viniferin. T*rans*-resveratrol, in particular, is one of the most interesting food bioactive compounds, being present, for example, in wine and grapes [26], with several reported bioactive properties [13,26,27] and with high commercial potential [9]. The content of this compound in the GSE was increased about 25 times compared to the raw grapevine shoots, i.e., from 1.1 mg/g grapevine shoots to 27.4 mg/g GSE, on a dry basis. This is remarkable since the content of *trans*-resveratrol in foods (grapes, chocolate, peanuts, etc.) is in the order of μg per g, and in wine, in the order of mg per L; hence, a dose probably too low to present therapeutical relevance in itself [26]. For example, in this work, two dosages of GSE were tested, 20 and 40 mg/kg bw, according to the preliminary toxicity study and a previous study on this animal model [27]. This means that about 0.5 and 1.1 mg/kg bw of *trans*-resveratrol was administered to the animals. Both GSE treatments reversed the effect of ISP on the animals (see Section 3 for the results, and the discussion below), but the second dose was more effective considering heart gene biomarker expression (Figure 5) and histology analysis (Figure 7). For a 60 kg human, the GSE dose could range from about 0.2 g to 0.4 g of GSE and about 5.5 to 11.0 mg of *trans*-resveratrol, respectively [15].

Moreover, *trans*-ε-viniferin (a dimeric form of resveratrol) and other derivatives of *trans*-resveratrol were found in GSE. These compounds are generated by oxidative polymerization of *trans*-resveratrol in the plant [28]. The content of stilbenoid oligomers depends on the plant part and cultivar, but the degree of polymerization is increased from the cane (mature and woody shoots) to the rootlets [6]. This agreed with the present results since GSE contained a relatively higher amount of stilbenoids in monomeric and dimeric forms than trimers and tetramers. These compounds can also contribute to the bioactivity of the extract along with other phenolic compounds like flavanols and gallic acid [27], or the *trans*-resveratrol metabolites generated in vivo after consumption [29].

Various studies have linked the presence of *trans*-resveratrol in wine to support the French Paradox and the lower incidence of cardiovascular events in that population [27,29]. Moreover, the potential protective role of *trans*-resveratrol on cardiovascular and heart health has also been evidenced [13]. Hence, in the present work, the possible therapeutic effect of GSE, which contained *trans*-resveratrol and derivatives (including *trans*-ε-viniferin), was investigated on cardiac remodeling by MI induction in Wistar rats with ISP. Indeed, MI is the most common source of demise in people suffering from cardiovascular illnesses.

Electrocardiographic abnormalities are the most used criterion for myocardial infarction diagnosis. In the current investigation, the increase in ST-segments observed following ISP treatment was corrected by the pre-co-treatment with GSE. It was clear that the administration of ISP significantly increased the levels of specific diagnostic markers (CK-MB, LDH, AST, and cTn-I), indicating impaired cardiac functions [30]. On the other hand, the pre-co-administration with GSE (20 or 40 mg/kg bw) reduced the amounts of the studied biomarkers. The beneficial effect of GSE was probably due to an improved integrity of the myocardial membrane, thus inhibiting the release of the aforementioned markers into the circulation [31]. The histopathological findings aligned with the biochemical markers, indicating a decrease in the edematous myocardium, without necrosis when animals were treated with GSE.

To further investigate the possible cardioprotective potential of GSE against ISP-induced injury, the serum lipid constituents were measured, namely, TC, TG, LDL-C, and HDL-C. The alteration in the lipid profile markers is considered a main risk factor in MI according to a previous study [32]. It was clear in this study that the pre-co-treatment with GSE nearly restored the elevated levels of plasmatic TC, LDL, and TG of ISP-induced MI in rats. Particularly, the observed cholesterol-lowering properties are probably due to the capacity of the biomolecules in GSE to inhibit key enzymes in cholesterol synthesis, like 3-hidroxi-3-metilglutaril-coenzima A (HMG-CoA) reductase [33]. Moreover, the use of GSE elevated the diminished levels of HDL-C observed in the ISP group, thus promoting cholesterol metabolism in the liver [34]. These findings agree with previous results regarding the capacity of *trans*-resveratrol to avoid dyslipidemia and myocardial infarction [35].

In addition, Figure 3a shows that the pre-co-administration of GSE with ISP showed an antithrombotic effect by reducing the plasma fibrinogen level. According to a previous study by Mnafgui et al. [36], the detected antithrombotic capacity of GSE might be influenced by the reduction in the protein disulfide isomerase (PDI), a protein involved in blood clotting. As another potential mechanism, a recent study suggests that *trans*-resveratrol can modulate the tissue factor (TF) (or thromboplastin) activity, concentration, and mRNA expression, while its metabolites can be deconjugated by endothelial cells and then also act by modulating the TF. The TF activates factor VII and triggers thrombosis [37].

The increased MDA levels and protein carbonyls associated with a decreased activity of the antioxidant enzyme system (e.g., SOD and CAT) may cause an increase in free radicals, which are responsible for oxidative stress, which in turn has been associated with MI development [38]. The results shown in Table 5 highlight a rise in the level of MDA when the animals were administered ISP, which was successfully reversed when the rats were treated with GSE. The reduced levels of this marker in GSE pre-co-treated rats could be a result of scavenging capacity against reactive oxygen species (ROS) carried out by natural products [39]. Particularly, *trans*-resveratrol can act against ROS in multiple ways, including by inhibiting their production and via the upregulation of antioxidant enzyme expression [35].

Furthermore, the groups that received GSE had better levels of CAT, SOD, and GSH, in comparison with the ISP group, suggesting that the acute toxicity induced by ISP was restored. Indeed, decreased levels of GSH, an important reducing agent in the cell, are associated with oxidative stress. These results confirmed one more time the antioxidant potency of GSE. It incited the antioxidant enzymes and restored the GSE level, ameliorating cellular protection against oxidative stress, and overall protecting the myocardium, even at lower dosages compared to other plant species extracts [40,41].

According to Table 5, ISP significantly elevated the plasmatic concentration of Ca^2+^, which could promote an excessive intracellular Ca^2+^ level in the cardiomyocytes [42]. The decreased level of the plasmatic Ca^2+^ following GSE pre-co-treatment seemed to be due to the natural molecules that can block the voltage-dependent L-type Ca^2+^ channels, according to a previous study [40]. This suggests the favorable impact of GSE on maintaining calcium homeostasis, potentially enhancing cardiac contractile function [43] and therefore avoiding MI [44]. In addition, the present work also evidenced that the pre-co-treatment with GSE improved the ionic homeostasis as confirmed by the increase in the level of Na^+^. The detected effect could be related to better membrane integrity and the normal function of the sodium–potassium pumps [36], and/or the inhibition of the renin–angiotensin system within the heart [45], in line with the ACE inhibition properties of the GSE.

The effect of ISP on the DNA structure was also targeted. The results of the DNA electrophoresis showed a laddered pattern of DNA fragments in ISP-treated rats in contrast to control animals. The GSE pre-co-treatment was able to decrease the observed changes in the DNA. The cardioprotective effects of GSE could be due to its antioxidative power and therefore the suppression of ROS-induced DNA damage [46].

Furthermore, Figure 3b evidenced the capacity of GSE to inhibit the ACE activity in the plasma of ISP-treated rats. It has been reported that the inhibition of ACE was associated with inhibited expression of the transforming growth factor (TGF)-β1, which is crucial in fibrosis. Hence, it could decrease the fibrotic process [23].

## 5. Conclusions

This current study offers, for the first time, experimental evidence on the effectiveness of the application of grapevine shoots as a source of biomolecules with cardio-preventive effects against ISP-induced acute MI and cardiac remodeling processes. It was remarkable that GSE enhanced the lipid profile and averted the altered concentration of calcium and sodium in plasma. It also provoked a notable decrease in markers of cardiac dysfunction, DNA fragmentation, and necrosis of the myocardial tissue. Furthermore, this study evidenced the anti-oxidative role of GSE, which increased the antioxidant enzymatic activity and decreased the lipid peroxidation in the heart. Additionally, GSE inhibited ACE activity that could be related to a potential anti-thrombotic effect. The results suggest that the GSE studied could serve to obtain stilbenoids, specifically *trans*-resveratrol and *trans*-ε-viniferin, for applications in the food and pharmaceutical industries to improve heart health and antioxidant status.

## Figures and Tables

**Table 1:** Contents of phenolic compounds (TPC), *trans*-resveratrol, *trans*-ε-viniferin, and the antioxidant activity of the grapevine shoot extract (GSE).

Component	Content
TPC (mg gallic acid eq./g GSE)	207.3 ± 3.5
*Trans*-resveratrol (mg/g GSE)	27.4 ± 0.3
*Trans*-ε-viniferin (mg/g GSE)	29.2 ± 0.2
FRAP (mg Trolox eq./g GSE)	137.58 ± 3.57

**Table 2:** Phenolic compounds extracted from grapevine shoots and characterized based on RP-HPLC-QTOF-MS analysis.

Proposed Compound	RT (min)	[M-H]- *m/z*	Molecular Formula	Score	Error (ppm)	Phenolic Class
Gallic acid	0.8	169.014	C_7_H_6_O_5_	98.9	−1.0	Hydroxybenzoic acid
Dihydroxybenzaldehyde	2.1	137.025	C_7_H_6_O_3_	99.2	−0.7	Phenol aldehyde
Hydroxybenzaldehyde	3.4	121.030	C_7_H_6_O_2_	99.7	−0.7	Phenol aldehyde
(Epi)catechin B-type dimer	4.4	577.135	C_30_H_26_O_12_	91.6	−1.3	Flavanol dimer
Catechin	4.4	289.073	C_15_H_14_O_6_	94.7	−3.0	Flavanol monomer
Epicatechin	6.5	289.072	C_15_H_14_O_6_	97.0	−2.0	Flavanol monomer
Oxidized resveratrol dimer 1	7.6	471.145	C_28_H_24_O_7_	98.4	0.5	Stilbenoid dimer
Oxidized resveratrol dimer 2	7.9	471.146	C_28_H_24_O_7_	96.3	−2.0	Stilbenoid dimer
Resveratrol hexoside	9.3	389.125	C_20_H_22_O_8_	96.0	−1.9	Stilbenoid monomer
Oxidized resveratrol dimer 3	9.4	471.145	C_28_H_24_O_7_	95.8	−1.2	Stilbenoid dimer
(Epi)catechin gallate	9.6	441.084	C_22_H_18_O_10_	97.7	−1.8	Flavanol monomer
Piceatannol	9.9	243.067	C_14_H_12_O_4_	96.7	−1.4	Stilbenoid monomer
Ampelopsin A	10.5	469.129	C_28_H_22_O_7_	97.5	−0.4	Stilbenoid dimer
*trans*-Resveratrol	12.3	227.072	C_14_H_12_O_3_	96.6	−2.0	Stilbenoid monomer
Pallidol	12.7	453.135	C_28_H_22_O_6_	97.1	−1.8	Stilbenoid dimer
Resveratrol isomer	14.0	227.072	C_14_H_12_O_3_	98.2	−0.7	Stilbenoid monomer
Viniferol E	15.5	923.270	C_56_H_44_O_13_	97.8	1.3	Stilbenoid tetramer
Hopeaphenol	16.1	905.260	C_56_H_42_O_12_	98.7	0.6	Stilbenoid tetramer
Viniferin isomer 1	16.6	453.135	C_28_H_22_O_6_	96.3	−1.3	Stilbenoid dimer
Hopeaphenol isomer	16.6	905.260	C_56_H_42_O_12_	94.0	0.0	Stilbenoid tetramer
Vitisinol C	17.2	427.155	C_27_H_24_O_5_	98.3	−0.4	Stilbenoid dimer
*trans*-ε-Viniferin	17.6	453.135	C_28_H_22_O_6_	96.6	−0.2	Stilbenoid dimer
Miyabenol C	18.8	679.198	C_42_H_32_O_9_	97.0	−0.6	Stilbenoid trimer
Viniferin isomer 2	18.9	453.135	C_28_H_22_O_6_	98.4	−1.4	Stilbenoid dimer
α-Viniferin	19.9	677.183	C_42_H_30_O_9_	90.0	−2.0	Stilbenoid trimer
Vitisin B isomer 1	23.2	905.259	C_56_H_42_O_12_	96.5	1.9	Stilbenoid tetramer
Vitisin B isomer 2	23.4	905.259	C_56_H_42_O_12_	98.3	1.3	Stilbenoid tetramer

**Table 3:** Other components of the grapevine shoot extract (GSE).

Component	Content
*Ultimate analysis (* *%, g/100 g GSE)*	
Carbon	52.87 ± 0.11
Hydrogen	6.69 ± 0.01
Nitrogen	1.06 ± 0.03
Sulfur	0.07 ± 0.09
*Other components (%, g/100 g GSE)*	
Moisture	9.24 ± 0.11
Ash	6.33 ± 0.08
*Other components (mg/g GSE)*	
Protein ^2^	66.3 ± 1.9
Oligomeric glucose	59.8 ± 4.4
Oligomeric galactose	3.1 ± 0.3
Monomeric glucose	55.7 ± 5.3
Monomeric galactose	5.0 ± 1.0
Mannitol	27.8 ± 2.3
Organic acids ^1^	38.9 ± 0.5

^1^ As acetic and formic acids. ^2^ Determined as nitrogen × 6.25.

**Table 4:** Rats’ body and heart weight, and cardiac weight index (CWI) of the control (C) and treated groups (mean ± SD, *n =* 6 rats per group).

Parameter	C	GSE1	GSE2	ISP	GSE1 + ISP	GSE2 + ISP
Body weight (g)	285.5 ± 2	291.8 ± 2.9	289.9 ± 0.9	282.7 ± 4.2	291.4 ± 2.1	286.4 ± 4.2
Heart weight (g)	0.73 ± 0.10	0.86 ± 0.05	0.80 ± 0.12	1.74 ± 0.10 ****	1.07 ± 0.11 ***	0.89 ± 0.10 ***
CWI	0.25 ± 0.02	0.29 ± 0.03	0.27 ± 0.03	0.61 ± 0.06 ****	0.36 ± 0.07 ***	0.31 ± 0.01 ***

GSE, grapevine shoot extract; ISP, isoproterenol. CWI: heart weight × 100/body weight. **** *p* < 0.0001: ISP vs. C; *** *p* < 0.001: GSE1 + ISP/GSE2 + ISP vs. ISP.

**Table 5:** Variation in the concentrations of lipids and studied electrolytes in plasma and thiobarbituric acid reactive substances (TBARS), enzymatic antioxidants, and total cellular glutathione (GSH) in the cardiac tissue of different experimental groups (mean ± SD, *n =* 6 rats per group).

Parameter	C	GSE1	GSE2	ISP	GSE1 + ISP	GSE2 + ISP
*Plasma lipids*						
TC (mg/dL)	67.6 ± 2.0	70.4 ± 3.6	70.8 ± 7.8	142.0 ± 6.5****	82.4 ± 9.9 ****	85.7 ± 4.0 ****
TG (mg/dL)	32.8 ± 3.6	30.5 ± 4.9	35.1 ± 4.6	75.4 ± 4.9 ****	46.3 ± 10.8 ****	40.4 ± 4.0 ****
LDL-C (mg/dL)	23.9 ± 2.5	28.8 ± 5.8	25.1 ± 7.0	98.9 ± 10.9 ****	52.2 ± 14.3 ****	40.0 ± 8.5 ****
HDL-C (mg/dL)	36.5 ± 6.1	32.4 ± 4.42	30.3 ± 2.8	17.8 ± 3.2 ****	29.0 ± 5.7 ***	30.5 ± 4.7 ***
*Plasma electrolytes*						
Na^+^ (mmol/L)	133.8 ± 2.3	130.2 ± 4.4	119.7 ± 19.7	90.97 ± 4.6 ****	117.1 ± 8.2 **	122.1 ± 12.7 ***
Ca^2+^ (mmol/L)	6.9 ± 1.0	6.9 ± 2.4	6.348 ± 4.1	14.4 ± 0.8 ****	8.7 ± 2.2 **	8.3 ± 1.5 ***
*TBARS and antioxidant status*						
TBARS (nmol MDA/g tissue)	0.79 ± 0.38	1.19 ± 0.76	1.27 ± 0.41	6.62 ± 0.71 ****	2.10 ± 1.02 ****	1.97 ± 0.93 ****
CAT (µmol destroyed H_2_O_2_/min per mg protein)	21.77 ± 3.38	21.28 ± 4.72	19.23 ± 4.95	7.11 ± 3.71 ****	15.65 ± 3.33 **	18.58 ± 2.41 ***
SOD (U/mg protein)	26.53 ± 3.14	24.07 ± 4.47	27.03 ± 4.89	11.85 ± 3.45 ****	20.90 ± 4.98 **	21.62 ± 4.09 **
GSH (U/mg protein)	6.32 ± 1.09	6.028 ± 0.43	6.34 ± 0.65	2.15 ± 0.55 ****	5.33 ± 0.61 ****	5.38 ± 1.01 ****

CAT, catalase; HDL-C, HDL-cholesterol; LDL-C, LDL-cholesterol; MDA, malondialdehyde; SOD, superoxide dismutase; TC, total cholesterol; TG, triglycerides. C, control; GSE, grapevine shoot extract; ISP, isoproterenol. **** *p* < 0.0001: ISP vs. C; ** *p* < 0.01, *** *p* < 0.001 or **** *p* < 0.0001: GSE1 + ISP/GSE2 + ISP vs. ISP.

## Data Availability

The data are contained within the article or Supplementary Materials.

## Supplementary Materials

The following supporting information can be downloaded at: https://www.mdpi.com/article/10.3390/foods12234351/s1, Table S1: Primer sequences used for semi-quantitative RT-PCR reactions; Figure S1: Electrocardiogram (ECG) patterns of the control and experimental animals. The control group showed a normal ECG pattern. The isoproterenol (ISP)-treated group showed pathological changes as ST-segment elevation (Pardee wave) (arrow).
