# Supplementary material for: Grapevine Shoot Extract Rich in Trans-Resveratrol and Trans-ε-Viniferin: Evaluation of Their Potential Use for Cardiac Health
Source: Foods. 2023 Dec 2;12(23):4351. doi: 10.3390/foods12234351 (PMC10705905; doi:10.3390/foods12234351)
Supplement: Supplementary file 1 [file foods-12-04351-s001.zip › foods-2718401-supplementary.pdf]

Table S1. Primer sequences used for semi-quantitative RT-PCR reactions.

Gene	Forward (5′-3′)	Reverse (5′-3′)
Bax	5'-CGGCGAATTGGAGATGAACTGG-3'	5'-CTAGCAAAGTAGAAGAGGGCAACC-3'
Caspase-3	5'-GTGGAACTGACGATGATATGGC-3'	5'-CGCAAAGTGACTGGATGAACC-3'
Bcl-2	5'-TGTGGATGACTGACTACCTGAACC-3'	5'-CAGCCAGGAGAAATCAAACAGAGG-3'

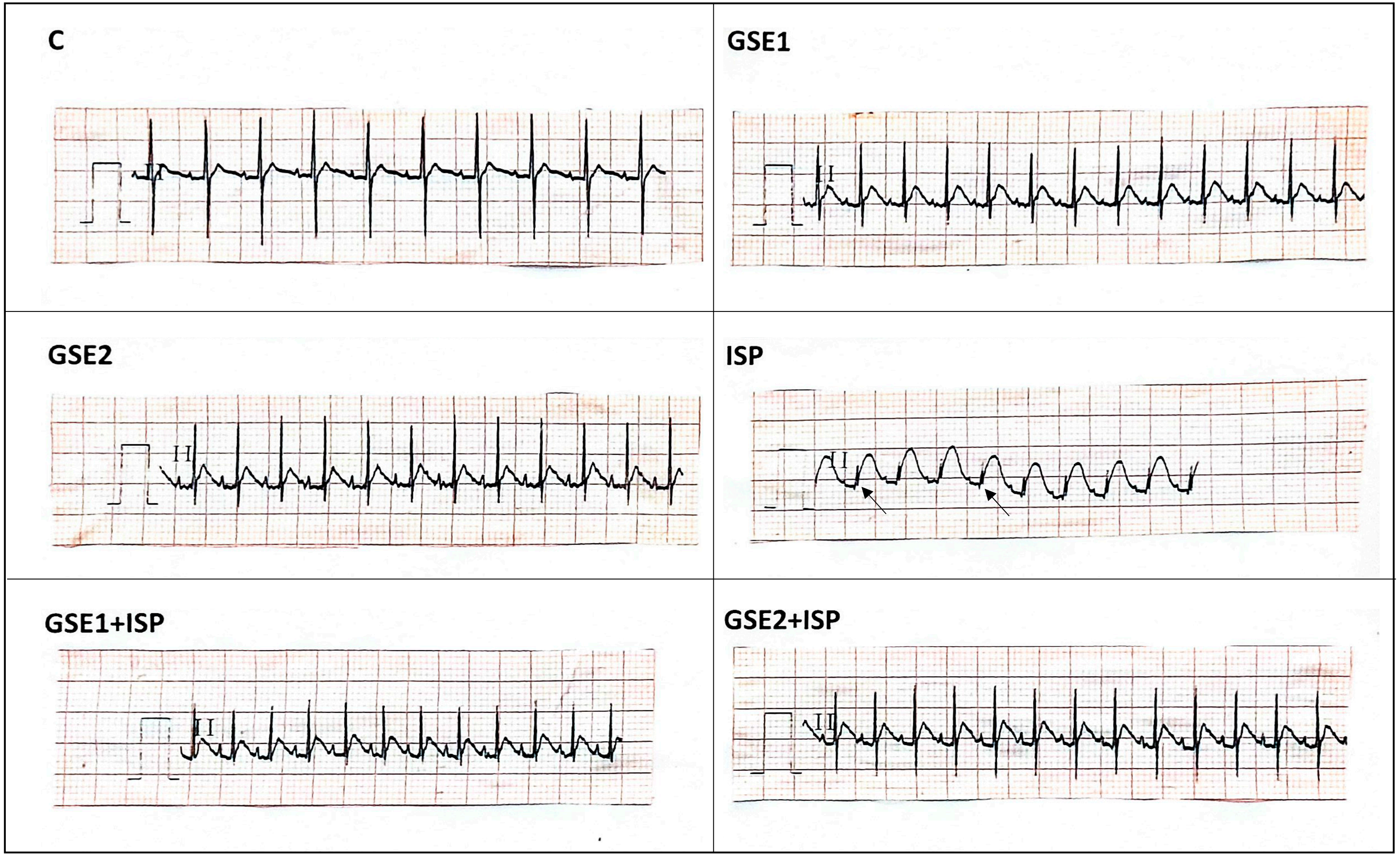

Figure S1. Electrocardiogram (ECG) pattern of control (C) and experimental animals. The control group showed a normal ECG pattern. The isoproterenol (ISP)-treated group showed pathological changes as ST-segment elevation (Pardee Wave) (arrow). GSE: grapevine shoot extract.
